# Concentrations of Bioelements (Zn, Cu, Fe, Cr, Mg, Mn) in Serum and Bone Tissue of Aging Men Undergoing Hip Arthroplasty: Implications for Erectile Dysfunction

**DOI:** 10.3390/biom14050565

**Published:** 2024-05-09

**Authors:** Aleksandra Rył, Żaneta Ciosek, Aleksandra Szylińska, Alina Jurewicz, Andrzej Bohatyrewicz, Iwona Rotter

**Affiliations:** 1 Department of Medical Rehabilitation and Clinical Physiotherapy, Pomeranian Medical University, Żołnierska 54b, 71-210 Szczecin, Poland; zaneta.ciosek@pum.edu.pl (Ż.C.); iwona.rotter@pum.edu.pl (I.R.); 2 Department of Cardiac Surgery, Pomeranian Medical University, al. Powstańców Wielkopolskich 72, 71-899 Szczecin, Poland; aleksandra.szylinska@pum.edu.pl; 3 Department of Specialized Nursing, Faculty of Health Sciences, Pomeranian Medical University, Żołnierska 48, 71-210 Szczecin, Poland; alina.jurewicz@pum.edu.pl; 4 Department of Orthopaedics, Traumatology and Orthopaedic Oncology, Pomeranian Medical University, Unii Lubelskiej 1, 71-252 Szczecin, Poland; andrzej.bohatyrewicz@pum.edu.pl

**Keywords:** erectile dysfunction, concentration of bioelements, serum, bone tissue, men

## Abstract

**Background:** Erectile dysfunction (ED) stands out as one of the most prevalent sexual disorders in men, with its incidence progressively escalating with age. As delineated by the International Consultation Committee for Sexual Medicine on Definitions/Epidemiology/Risk Factors for Sexual Dysfunction, the prevalence of ED among men under 40 years is estimated to be within the range of 1–10%. The aim of this study was to determine the relationship between the concentration of bioelements (Zn, Cu, Fe, Cr, Mg, and Mn) in the serum and bone tissue and the concentration of selected hormones in men with and without erectile dysfunction. **Materials and methods:** The retrospective cohort study included 152 men who underwent total hip arthroplasty for hip osteoarthritis at the Department of Orthopaedic Traumatology and Musculoskeletal Oncology at the Pomeranian Medical University in Szczecin. Certain exclusion criteria were applied to ensure the integrity of the study. These included individuals with diabetes, a history of cancer, alcohol abuse, liver or kidney failure, New York Heart Association (NYHA) class III or IV heart failure, and those taking medications that affect bone metabolism, such as mineral supplements, neuroleptics, chemotherapeutic agents, immunosuppressants, corticosteroids, or antidepressants. Patients with hypogonadism or infertility were excluded from the study. **Results**: The study showed an association between bioT concentrations and Cu concentrations in both patients with and without erectile dysfunction. A correlation between bioactive testosterone and Cr concentrations was also observed in both groups. Patients with erectile dysfunction showed a relationship between bioT concentration and Zn concentration, TT concentration and Mn concentration, FT concentration and Zn concentration, and E2 concentration and Cr concentration. An analysis of elemental concentrations in bone tissue showed an association between FT and Mg and Mn concentrations, but only in patients with erectile dysfunction. In patients without erectile dysfunction, a correlation was observed between FT and Cu concentrations. A correlation was also observed between bioT concentrations and Mg, Mn, and Zn concentrations, but only in patients with erectile dysfunction. In patients without erectile dysfunction, a correlation was observed between bioT and Cu concentrations. **Conclusions**: Studying the relationship between the concentration of bioelements (Zn, Cu, Fe, Cr, Mg, and Mn) in the serum and bone tissue and the concentration of selected hormones in men may be important in explaining the etiology of the problem. The study of the concentration of Zn and Cu in bone tissue and serum showed that these two elements, regardless of the place of accumulation, may be related to the concentration of androgens in men.

## 1. Introduction

Erectile dysfunction (ED) stands out as one of the most prevalent sexual disorders in men, with its incidence progressively escalating with age. As delineated by the International Consultation Committee for Sexual Medicine on Definitions/Epidemiology/Risk Factors for Sexual Dysfunction, the prevalence of ED among men under 40 years is estimated to be within the range of 1–10%. This percentage increases to 20–40% for men aged 60–69 years and reaches 50–100% for those over 70 years [1,2]. Projections suggest that the global count of ED patients will hit 322 million by 2025 [3].

The equilibrium of trace elements within the human body serves as a fundamental prerequisite for normal physiological functioning. Trace metals actively participate in averting vascular damage and atherosclerosis through various pathways, thereby safeguarding the relatively narrow-diameter penile arteries. Existing studies (including multiple animal studies) propose that adhering to recommended dietary metal intake limits may prove advantageous in reducing the prevalence of erectile dysfunction, although research on the correlation between dietary metal intake and ED prevalence remains limited [4].

Zinc (Zn), an essential trace mineral, plays a pivotal role in the metabolic activity of 300 enzymes, facilitating cell division, DNA, and protein synthesis [5]. It sustains optimal testosterone levels [6,7], potentially suppressing inflammation [8] and preserving penile endothelial function. This preservation promotes erection and male sexual activity [9]. In individuals of sound health, fasting plasma Zn concentrations are homeostatically maintained within narrow limits (approximately 80 to 100 µg/dL or 12 to 15 µmol/L) [10]. Zn deficiency, and, consequently, low testosterone levels, can lead to erectile dysfunction and compromised orgasm control. Testosterone, a hormone pivotal in male sexual health, influences sexual organ development, sex drive, and sperm production, and its insufficient levels correlate with erectile dysfunction, diminished libido, and infertility [11]. Moreover, deficient androgenic hormone levels impair sperm production, potentially resulting in infertility. Inadequate Zn levels in the body negatively impact spermatogenesis processes, affecting sperm formation and maturation, leading to reduced sperm count and viability [12]. Testosterone in the blood occurs in the free form (1–4%), bound to albumin (less than 44%), and bound to the SHBG transport protein (approx. 55%). Free testosterone affects target tissues after binding to intracellular receptors that activate specific genes. As a consequence, it causes an increase in the synthesis of appropriate proteins in target tissues (anabolic effect). Sex-hormone-binding globulin (SHBG) transports testosterone within the bloodstream and regulates its bioavailability and access to extravascular target tissues [13]. In men, plasma testosterone levels fluctuate throughout life and begin to decrease in middle age and continue to decline with age [14,15].

Copper (Cu), another essential mineral, assumes a critical role in numerous bodily functions [16]. It is integral for red blood cell formation and hemoglobin synthesis, the protein responsible for oxygen transport in the blood. Cu also influences androgen metabolism at the hypothalamic–pituitary–gonadal axis, which is crucial for fertility. In blood serum, Cu remains at a steady level of approximately 1 mg/L, with 95% bound to ceruloplasmin, 3–5% bound to albumin and transcuprein, and 1% bound to low-molecular-weight amino acids and peptides [17]. Research indicates that insufficient Cu levels may contribute to ED through decreased testosterone levels in men [18]. Cu actively participates in nitric oxide production, a compound that facilitates blood vessel relaxation and increased blood flow to the penis. Inadequate Cu levels may impede nitric oxide production, resulting in reduced blood flow and difficulties in achieving or maintaining an erection [4].

Iron (Fe), a component of hemoglobin, a protein found in red blood cells responsible for oxygen transport, plays a vital role in sexual health. It is also present in crucial enzymes involved in DNA formation, neurotransmitter synthesis in the brain, and cellular energy storage and processing [19]. Normal Fe concentrations in men range from 17.7 to 35.9 µmol/L (90–200 µg/dL) [20]. It is instrumental in ensuring adequate blood flow to the genitals. Sufficient Fe enables the production of hemoglobin, facilitating oxygen-rich blood flow to the genitals, promoting sexual arousal, and increasing sensitivity. Insufficient Fe levels can result in sexual dysfunction, including decreased libido and difficulties in achieving or maintaining an erection. This is attributed to the body’s inability to produce enough hemoglobin for oxygen transport to the genitals, leading to diminished blood flow and sensitivity [21].

Chromium (Cr), a micronutrient with multifaceted functions in the body, is a vital component of the glucose tolerance factor, actively participating in glucose metabolism [22]. The average Cr levels in serum and urine for the general population are approximately 0.10–0.16 and 0.22 μg/L, respectively. The two isotopes of Cr, trivalent and hexavalent, exert distinct effects on the human body [22]. Notably, Cr may induce reproductive toxicity in the male reproductive system, leading to decreased sperm count and motility or an increase in abnormal sperm [23]. The testes’ development, morphology, and function are significantly impacted, resulting in reduced male fertility [24]. Cr-induced reproductive toxicity is attributed to altered sex hormone secretion and oxidative stress. The accumulation of Cr in the testes disrupts the blood–testis barrier, affecting Sertoli cells’ normal function. This disruption increases FSH levels and decreases testosterone levels, directly influencing sperm count and potentially contributing to erectile dysfunction. Oxidative stress induced by Cr emerges as a significant factor in male infertility [25].

Magnesium (Mg), an essential mineral crucial for normal human development and function, maintains a normal concentration in human blood serum ranging from 0.75 to 0.95 mmol/L (1.8–2.3 mg/dL, or, in a wider range, 0.65–1.25 mmol/L) [26]. Approximately 55–70% of Mg exists in ionized form, with the remainder being bound, primarily with albumin [27,28,29,30]. Mg plays a pivotal role in several physiological processes impacting sexual health. Firstly, it enhances blood flow to the penis, a critical factor for achieving and maintaining an erection [31]. This effect is achieved by regulating the body’s nitric oxide (NO) levels, a compound that relaxes blood vessels and promotes blood flow. Secondly, Mg aids in stress and anxiety reduction, known for improving mood and energy levels essential for a healthy sex life [31]. Thirdly, Mg is integral to the production of sex hormones, such as testosterone and estrogen, responsible for regulating sexual desire and function in men. Additionally, it exhibits positive effects on overall cardiovascular health, a crucial aspect of sexual function due to the heart and blood vessels’ role [32].

Manganese (Mn), a trace mineral with diverse biological roles, including energy production, collagen synthesis, and immunity, maintains normal serum levels in the range of 0.4–0.85 μg/L [33]. Recent studies suggest Mn’s potential impact on the male erection. It is essential for nitric oxide (NO) production, facilitating blood vessel relaxation and increased blood flow to the penis. Mn may also play a role in testosterone production, a key sex hormone for normal sexual function. Supplementation with Mn may maintain thiol concentrations, reduce oxidative stress in human sperm, and improve sperm quality and motility by activating adenylate cyclase activity [34].

In the existing literature, limited evidence associates erectile dysfunction with serum and bone tissue concentrations of bioelements. This study marks the first comprehensive analysis of the relationship between erectile dysfunction in men and the concentration of bioelements (Zn, Cu, Fe, Cr, Mg, and Mn) in both serum and bone tissue.

The aim of the study was to determine the relationship between the concentration of bioelements (Zn, Cu, Fe, Cr, Mg, and Mn) in the serum and bone tissue and the concentration of selected hormones in men with and without erectile dysfunction.

## 2. Materials and Methods

### 2.1. Study Participants

The study included 152 men who underwent total hip arthroplasty for hip osteoarthritis at the Department of Orthopaedic Traumatology and Musculoskeletal Oncology at the Pomeranian Medical University in Szczecin. Certain exclusion criteria were applied to ensure the integrity of the study. Patients with diabetes, a history of cancer, alcohol abuse, liver or kidney failure, New York Heart Association (NYHA) class III or IV heart failure, and those taking medications that affect bone metabolism, such as mineral supplements, neuroleptics, chemotherapeutic agents, immunosuppressants, corticosteroids, or antidepressants, were excluded. Patients who reported conditions related to hypogonadism and a history of infertility were also excluded.

### 2.2. Division into Groups

The men were asked to complete an abbreviated questionnaire with sociodemographic data and an abbreviated version of the International Index of Erectile Function (IIEF) (five items). This questionnaire is designed for the self-assessment of sexual function over the past 4 weeks. It allows differentiation of erectile dysfunction in the areas of erectile dysfunction, reaching orgasm, sexual desire, satisfaction with sexual life, and overall sexual satisfaction [35]. A score of 21 and below qualified for the ED group, and a score above 21 qualified for no ED group.

### 2.3. Measurement of Sex Hormones

Venous blood samples were collected from all participants after an overnight fast (between 07:00 and 09:00) and stored at −20 °C until further processing. The concentrations of total testosterone (TT, normal range for men: 2.36–9.96 ng/mL), estradiol (E2, normal range for men: 11.2–50.4 pg/mL), sex-hormone-binding globulin (SHBG, normal range: 18–110 nmol/L), dehydroepiandrosterone sulfate (DHEAS, normal range: 110–470 μg/dL), and insulin (I, normal range: 5–25 μIU/mL) were determined using ELISA assays (DRG Medtek, Warsaw, Poland). Free testosterone (FT, normal range: 8.9–45.5 pg/mL) levels were calculated according to the formula developed by Vermeulen: FT = (TT − N − SHBG + √((N + SHBG − TT)2 + 4NT))/2N, where N = 0.5217 × albumin concentration + 1 [36]. Bioavailable testosterone (bioT) was calculated using the formula developed by Morris et al. [37].

The lipid accumulation product (LAP) was calculated according to the following formula: LAP = (WC (cm) − 65) × TAG (mmol/L) [38]. Visceral adiposity index (VAI) was calculated using the following formula: VAI = WC (cm)/[39.68 + (1.88 × BMI)] × ((TAG/1.03) × (1.31/HDL-C)) [39]. Body mass index (BMI) and waist-to-hip ratio (WHR) were calculated. HOMA-IR was calculated using the following formula: fasting insulin (microU/L) x fasting glucose (nmol/L)/22.5.

The following markers of bone turnover were measured: osteocalcin (OC, normal range: 5–25 ng/mL), parathyroid hormone (PTH, normal range: 10–60 pg/mL), carboxy-terminal collagen I crosslinks (CTX-I, normal range: 0.115–0.748 ng/mL), and procollagen type I N-terminal propeptide (PINP, normal range: 85.55–2028.75 ng/mL).

### 2.4. Measurement of Bioelements

Serum and bone samples were stored at −80 °C until analysis. Serum magnesium (Mg), calcium (Ca), and phosphorus (P) concentrations were determined by inductively coupled plasma optical emission spectrometry (iCAP™ 7400 ICP-OES Analyzer; Thermo Fisher Scientific, Waltham, MA, USA). This technique is widely accepted and powerful for the analysis and quantification of trace elements in both liquid and solid samples. Analysis was performed in both radial and axial modes.

The serum samples were thawed at room temperature and digested using the CEM MARS 5 oven digestion system. The bone samples were also thawed at room temperature and dried at 80 °C overnight until a constant weight was reached after the removal of any adherent tissue. The bones were ground to powder using a porcelain mortar and mineralized using the CEM MARS 5 system. A minimum of 0.1 g of bone tissue was then allowed to pre-react for 30 min in a clean hood. After the pre-reaction, 1 mL of unstabilized 30% H_2_O_2_ was added. The samples were placed in special Teflon containers and heated in a microwave digestion system at 180 °C for 35 min. After digestion, the samples were removed from the microwave and allowed to cool to room temperature. Blank samples were prepared by adding concentrated nitric acid to tubes without samples, and these blanks were then diluted in the same manner. Multi-element calibration standards (ICP multi-element standard solution IV, Merck, Darmstadt, Germany) with different concentrations of inorganic elements were prepared in the same way as the blanks and samples. Deionized water (Direct Q UV, Merck, Darmstadt, Germany, approximately 18.0 MΩ) was used for the preparation of all solutions. A Merck (Darmstadt, Germany) calibration standard consisting of steamed bone meal, which was sieved and mixed to ensure high homogeneity, was used to calibrate the bone samples. Quality control for the determination of trace elements in serum was performed by analysis of the SRM (8414 NIST Bovine muscle, Gaithersburg, MD, USA) using instrumental neutron activation analysis.

### 2.5. Statistical Analysis

Quantitative variables were presented as median, standard deviation (SD), lower quartile, and upper quartile. The normality of the data was assessed using the Shapiro–Wilk test. For normally distributed data, means were compared using a Student’s *t*-test. For non-normally distributed data, the non-parametric Mann–Whitney U test was used. Linear logistic regression analysis was also performed, adjusted for age and total weight.

The relationship expressed as the ratio of element concentrations in serum and bone tissue was also analyzed. For one analysis, the participants were divided into groups based on the median values of serum and bone Mg concentrations. These median values were 24.981 mg/L for serum concentration and 3705.193 mg/kg for bone concentration.

Statistical analyses were performed using Statistica software (version 12.0, StatSoft Poland, Krakow, Poland). The significance level was set at *p* ≤ 0.05.

## 3. Results

For statistical analysis, the first step was to compare a group of patients who were diagnosed with erectile dysfunction on the International Index of Erectile Function (IIEF) questionnaire with those who were not. The study showed that 109 patients had erectile dysfunction and 43 patients did not. This analysis showed that the patients in the two groups differed in their testosterone concentrations. When the concentrations of other hormones, as well as serum and bone tissue elemental concentrations, were analyzed, there were no statistically significant differences between the two groups analyzed (Table 1). It was shown that there was a tangential relationship between the study groups in the analysis of TT concentration (*p* = 0.009).

Table 2 and Table 3 show the linear regression analysis between the concentration of different fractions of testosterone and E2 and the concentration of bioT in serum (Table 2) and bone tissue (Table 3).

The study demonstrated an association between bioT concentrations and Cu concentrations in patients with (*p* = 0.019, beta = 0.325) and without erectile dysfunction (*p* = 0.049, beta = −0.393). Additionally, a correlation between bioactive testosterone and Cr concentrations was observed in both groups. Patients with erectile dysfunction demonstrated a correlation between bioT concentration and Cu concentration (*p* = 0.019, beta = −0.289), TT concentration and Mn concentration (*p* = 0.031, beta = −0.349), TT concentration and Cr concentration (*p* = 0.025, beta = −0.344), FT concentration and Zn concentration (*p* = 0.023, beta = −0.316), Cu concentration (*p* = 0.009, beta = −0.335) and Cr concentration (*p* = 0.012, beta = −0.373) and E2 concentration with Cr concentration (*p* = 0.001, beta = 0.477), Fe concentration (*p* = 0.028, beta = −0.263) and Mg concentration (*p* = 0.041, beta = 0.243).

In the group of patients without erectile dysfunction, a relationship was observed between FT concentration and Cr concentration (*p* = 0.033, beta = 0.711) and Cu concentration (*p* = 0.054, beta = −0.493) and between E2 concentration and Mg concentration (*p* = 0.009, beta = 0.984). In both groups, a relationship was observed between FT concentration and serum Cu concentration.

The analysis of elemental concentrations in bone tissue revealed an association between FT and Mg (*p* = 0.003, beta = −0.520) and Mn (*p* = 0.001, beta = 0.937) concentrations, Zn (*p* = 0.012, beta = 0.435) concentrations, Fe (*p* = 0.005, beta = −0.660) and Cr (*p* = 0.673, beta = −0.937) concentrations, and an association between BioT and Mg (*p* = 0.002, beta = −0.543) and Mn (*p* = 0.008, beta = 0.460) concentrations, Fe (*p* = 0.006, beta = −0.647) concentrations, and Cr (*p* = 0.027, beta = −0.696) concentrations, but only in patients with erectile dysfunction. In patients without erectile dysfunction, a correlation was observed between TT and Cu concentrations (*p* = 0.043, beta = 3.876) and Cr (*p* = 0.037, beta = −4.157) concentration.

## 4. Discussion

Erection stands as a fundamental aspect of male sexual function, and any disruption in this process can lead to both physical and psychological distress, garnering increasing scientific interest in recent years to identify the contributing factors [40]. One potential cause under scrutiny is the abnormal concentration of trace elements in the body.

Testosterone, a hormone pivotal in men’s sexual health, experiences a natural decline with age, with low levels linked to erectile dysfunction. This study revealed distinctions in testosterone levels between patients diagnosed with erectile dysfunction and those without such a diagnosis. Furthermore, a correlation was observed between bioavailable testosterone (bioT) levels and Zn, Cu, and Cr levels, as well as free testosterone (FT) levels and Cr levels among patients with erectile dysfunction. Prasad et al.’s study demonstrated a clear relationship between Zn and testosterone levels in 40 normal men aged 20 to 80 years. The study included normal young men undergoing dietary Zn restriction, resulting in a significant decrease in serum testosterone concentrations. Conversely, mildly Zn-deficient normal elderly men experienced an increase in serum testosterone levels after six months of Zn supplementation [41].

Besong et al. and Rafique et al. reached similar conclusions regarding the positive effect of Zn on maintaining optimal testosterone levels. Additionally, Trumbo et al.’s study suggests that increasing dietary Zn intake within the recommended range may be beneficial in reducing the risk of erectile dysfunction [7,42,43]. An animal study conducted in 2009 demonstrated that rats receiving 5 milligrams per day of Zn supplementation exhibited improved sexual function, with similar positive effects observed in men, emphasizing the potential of Zn in arousal and erection maintenance [44].

The precise mechanism through which Cu impacts erection remains incompletely understood, but various potential mechanisms have been proposed. Animal studies indicate that Cu deficiency may cause damage to the endothelium of blood vessels, potentially disrupting the normal blood supply to the penis [45]. In a rat study conducted by Chattopadhyay et al. in 2005, Cu demonstrated a dose-dependent effect on testicular steroidogenesis and spermatogenesis, influencing serum testosterone, FSH, and LH levels [46]. Tvrda et al. found that elevated Cu concentrations adversely affect sperm function, quality, motility, and mitochondrial activity [47]. Another study found a positive correlation between seminal plasma Cu concentration and sperm parameters, including volume and motility [48]. However, Seven et al. discovered that high Cu concentrations (≥100 μg/mL) were detrimental to sperm motility, morphology, and DNA [49].

The present study revealed a relationship between bioavailable testosterone (bioT) concentrations and Cu concentrations in patients with erectile dysfunction. Other studies support the close association between Cu levels and male sexual function. A study on 500 patients diagnosed with erectile dysfunction found that 80% had reduced Cu levels in their blood [50]. Additionally, research by Johnson et al. indicated that Cu deficiency interferes with the normal production and function of nitric oxide, a crucial factor in maintaining an erection [51]. The results from a multi-year study on the relationship between Cu levels and erection in men yielded conflicting results, with one study of 1000 men showing no significant correlation between Cu levels and the incidence of erectile dysfunction [52]. In contrast, a clinical study of 300 men diagnosed with erectile dysfunction found significant Cu deficiency in 85% of subjects [53]. These conflicting outcomes highlight the need for further research to elucidate the role of Cu in relation to erectile function in men.

Fe’s essential role in the production of red blood cells, carrying oxygen to body tissues, including the penis, underscores its significance for sexual health. Fe deficiency can lead to anemia, potentially reducing blood flow to the penis and impeding the ability to achieve an erection. A 2019 study found higher red cell distribution width (RDW) levels in men with erectile dysfunction compared to the control group, suggesting that RDW values may be a useful predictor for identifying and monitoring ED severity [54]. Some studies also propose that severely low testosterone levels are a common finding in cases of severe Fe overload, potentially contributing to erectile dysfunction [55]. However, the analysis of hormone levels and serum and bone tissue Fe concentrations in the present study did not reveal statistically significant differences between patients with and without erectile dysfunction.

Chromium exerts multiple influences on erection. Firstly, it plays a crucial role in glucose metabolism, a process vital for providing energy to penile muscles. Proper glucose metabolism is essential for maintaining normal sexual function. Secondly, Cr is involved in insulin production, a hormone regulating blood sugar levels. Men with insulin deficiency have an increased risk of erectile dysfunction. Thirdly, Cr contributes to maintaining normal cholesterol levels, crucial for averting heart disease and associated erectile problems. Abnormal Cr levels may also impact male infertility. This study’s results suggest correlations between bioactive testosterone and Cr concentrations, as well as free testosterone and serum Cu concentrations in the two groups analyzed. Additionally, patients with erectile dysfunction exhibited a correlation between estradiol levels and Cr levels. In comparison, Li et al. conducted a study on workers exposed to Cr (VI) and found significantly lower sperm counts and motility compared to an unexposed group [25]. Similar findings were reported by other authors investigating the fertility potential of men exposed to Cr (VI) [56,57].

Hypomagnesemia may contribute to erectile dysfunction through its association with decreased nitric oxide (NO), a crucial factor in initiating and maintaining erections. NO, key to endothelial function, requires Mg for synthesis. Hypomagnesemia decreases NO levels, leading to penile vasoconstriction and reduced blood flow. Mg deficiency also affects testosterone production. In this study, patients without erectile dysfunction showed a correlation between estradiol concentration and Mg concentration. The analysis of bone tissue concentration in patients with erectile dysfunction revealed a relationship between free testosterone concentration and Mg concentration, and bioavailable testosterone concentration and Mg concentration. Some studies suggest the role of decreased seminal Mg in premature ejaculation [58]. In contrast, a study by Volpe et al. proposed that increased dietary Mg intake below the recommended upper limit may reduce the risk of penile artery calcification and the prevalence of erectile dysfunction [59].

Manganese, a trace element involved in various biological processes, has garnered attention for its potential impact on erectile function. The study found correlations between total testosterone, bioavailable testosterone, and free testosterone concentrations in bone tissue and Mn concentrations in patients with erectile dysfunction. Similar studies by Sheng et al. revealed that men with low blood Mn levels had a significantly higher risk of erectile dysfunction. Participants with low Mn levels receiving a Mn supplement showed improvements in sexual function, including erections [60,61]. Avila reported an association between high Mn levels and erectile dysfunction [62].

In summary, both the deficiency and excess of trace elements in the male body can negatively affect the ability to maintain an erection. However, further research is needed to better understand the mechanisms of action and the exact relationship between trace element levels and erectile function. The recognition of trace elements’ impact on erection opens up new perspectives for the treatment of erectile dysfunction.

This study was limited in several ways. The main limitation was that it only involved men between the ages of 60 and 75. To obtain a comprehensive view of the relationship analyzed in our study, it would have been beneficial to include older patients. In addition, a more diverse patient cohort could enhance the generalizability of the findings. The exclusion of patients with conditions such as diabetes and cardiovascular diseases, which are prevalent in men with ED, may have limited our understanding of the interrelationship between these conditions and bioelement concentrations. Another limitation is the fact that hormone levels were determined using the ELISA method, which may have resulted in inaccurate measurements. It would be preferable to determine hormones using mass spectrometry. Additionally, the exclusion of patients with conditions such as diabetes and cardiovascular diseases, which are prevalent in men with ED, may have limited the ability to understand the interplay between these conditions and bioelement concentrations. Another limitation of the study was that all patients underwent arthroplasty. This is a very stressful situation for the patient and may result in the occurrence of functional hypogonadism.

The multifactorial nature of erectile dysfunction in men requires deeper research. Studying the relationship between the concentration of bioelements (Zn, Cu, Fe, Cr, Mg, and Mn) in the serum and bone tissue and the concentration of selected hormones in men may be important in explaining the etiology of the problem. In the study, we showed that there is a relationship between the concentration of bioelements and the concentration of hormones depending on the diagnosis of erectile dysfunction. However, the accumulation of these elements in different tissues varies. The study of the concentration of Zn and Cu in bone tissue and serum showed that these two elements, regardless of the place of accumulation, may be related to the concentration of androgens in men.

## Figures and Tables

**Table 1:** Descriptive statistical data.

		Patients without Erectile Dysfunction				Patients with Erectile Dysfunction				*p*
Variable		X	Minimum	Maximum	SD	X	Minimum	Maximum	SD	
**Age**		62.86	60.00	72.00	3.68	65.76	60.00	75.00	4.32	0.068
**BMI**		30.03	21.91	37.89	4.32	29.44	14.99	42.97	4.24	0.695
**IIEF**		22.86	22.00	25.00	1.03	15.94	5.00	21.00	3.85	<0.001 *
**Serum levels**	TT ng/mL	6.03	3.68	8.53	1.58	4.86	1.38	12.90	2.27	0.009 *
	FT ng/mL	0.09	0.03	0.16	0.04	0.09	0.02	0.23	0.04	0.634
	BioT ng/dL	2.12	0.97	3.87	0.85	1.94	0.42	5.06	0.93	0.411
	E2 pg/mL	88.04	20.92	170.83	37.31	83.96	28.26	180.53	36.29	0.569
	Zn	1.29	1.07	1.99	0.24	1.43	0.23	3.04	0.44	0.320
	Cu	1.00	0.76	1.40	0.16	0.97	0.25	1.45	0.18	0.844
	Fe	1.82	0.94	3.05	0.58	1.52	0.32	3.45	0.51	0.062
	Cr	0.008	0.002	0.01	0.003	0.01	0.006	0.002	0.001	0.222
	Mg	24.86	19.07	30.63	3.08	26.25	16.95	68.24	6.98	0.785
	Mn	0.01	0.01	0.01	0.00	0.01	0.00	0.02	0.00	0.086
**Bone levels**	Mg	3459.61	1501.77	5330.19	1299.42	3812.53	1220.72	10,255.82	1610.36	0.644
	Mn	0.27	0.05	0.88	0.26	0.62	0.07	5.08	0.95	0.142
	Zn	185.34	82.08	333.76	71.02	193.93	68.08	306.45	47.66	0.543
	Cu	1.42	0.17	4.19	1.02	1.51	0.23	7.45	1.11	0.906
	Fe	97.55	6.89	307.47	81.16	146.43	0.59	1112.01	193.71	0.531
	Cr	1.85	0.20	10.67	2.95	4.05	0.07	101.00	15.10	0.694

X—mean; SD—standard deviation; *p*—static significance; *—statistically significant parameter; BMI—body mass index; IIEF—International Index of Erectile Function; TT—total testosterone; FT—free testosterone; BioT—bioactive testosterone; E2—estradiol; Zn—zinc; Cu—copper; Fe—iron; Cr—chrome; Mg—magnesium; Mn—manganese.

**Table 2:** Concentrations of bioelements in serum.

Effect		Patients without Erectile Dysfunction				Patients with Erectile Dysfunction			
		*p*	−95.00% CI	+95.00% CI	Beta	*p*	−95.00% CI	+95.00% CI	Beta
**BioT**	Zn	0.049 *	−3.730	0.922	−0.393	0.019 *	0.113	1.229	0.325
	Cu	0.102	−5.531	0.625	−0.473	0.019 *	−2.820	−0.268	−0.298
	Fe	0.666	−1.248	0.848	−0.136	0.453	−0.585	0.265	−0.089
	Cr	0.050 *	−15.362	383.479	0.692	0.007 *	42.650	258.414	0.401
	Mg	0.913	−0.188	0.207	0.034	0.112	−0.056	0.006	−0.190
	Mn	0.778	−404.126	314.890	−0.091	0.107	−148.207	14.932	−0.248
**TT**	Zn	0.066	−7.622	0.325	−0.548	0.171	−0.444	2.437	0.196
	Cu	0.199	−2.104	8.414	0.327	0.168	−5.554	0.988	−0.181
	Fe	0.791	−1.582	2.000	0.076	0.374	−1.601	0.612	−0.111
	Cr	0.151	−108.659	572.781	0.468	0.025 *	40.784	598.937	0.344
	Mg	0.027 *	0.062	0.738	0.782	0.300	−0.123	0.039	−0.129
	Mn	0.479	−808.340	420.138	−0.211	0.031 *	−446.563	−22.025	−0.349
**FT**	Zn	0.131	−0.150	0.024	−0.400	0.023 *	0.004	0.053	0.316
	Cu	0.054 *	−0.228	0.002	−0.493	0.009 *	−0.133	−0.020	−0.335
	Fe	0.611	−0.048	0.030	−0.137	0.335	−0.028	0.010	−0.115
	Cr	0.033 *	0.878	15.822	0.711	0.012 *	1.409	10.931	0.373
	Mg	0.866	−0.008	0.007	−0.045	0.137	−0.002	0.000	−0.178
	Mn	0.760	−15.278	11.663	−0.083	0.187	−5.997	1.203	−0.202
**E2**	Zn	0.253	−140.542	43.543	−0.308	0.961	−21.902	20.867	−0.006
	Cu	0.441	−163.902	79.739	−0.184	0.474	−66.894	31.507	−0.087
	Fe	0.691	−48.768	34.205	−0.113	0.028 *	−34.897	−2.041	−0.263
	Cr	0.490	−5462.650	10,322.246	0.207	0.001 *	2878.157	11,154.738	0.477
	Mg	0.009 *	4.074	19.737	0.984	0.041 *	0.051	2.465	0.243
	Mn	0.695	−16,686.765	11,769.718	−0.113	0.622	−3933.646	2372.927	−0.074

*p*—statistical significance, *—statistically significant parameter, CI—confidence interval; BMI—body mass index, IIEF—International Index of Erectile Function, TT—total testosterone; FT—free testosterone; BioT—bioactive testosterone; E2—estradiol; Zn—zinc; Cu—copper; Fe—iron; Cr—chrome; Mg—magnesium; Mn—manganese.

**Table 3:** Concentrations of bioelements in bone.

Effect		Patients without Erectile Dysfunction				Patients with Erectile Dysfunction			
		*p*	−95.00% CI	+95.00% CI	Beta	*p*	−95.00% CI	+95.00% CI	Beta
**TT**	Mg	0.078	−0.009	0.001	−3.326	0.086	−0.002	0.000	−0.370
	Mn	0.300	−14.177	5.679	−0.668	0.477	−1.372	2.874	0.292
	Zn	0.072	−0.008	0.129	2.716	0.266	−0.010	0.035	0.231
	Cu	0.043 *	0.327	12.129	3.876	0.897	−1.694	1.490	−0.050
	Fe	0.958	−0.042	0.041	−0.041	0.697	−0.012	0.008	−0.161
	Cr	0.037 *	−4.337	−0.228	−4.157	0.988	−0.482	0.489	0.006
**FT**	Mg	0.985	0.000	0.000	0.051	0.003 *	0.000	0.000	−0.520
	Mn	0.385	−0.260	0.541	0.980	0.001 *	0.032	0.094	0.937
	Zn	0.940	−0.003	0.003	−0.159	0.012 *	0.000	0.001	0.435
	Cu	0.281	−0.131	0.345	2.945	0.230	−0.009	0.036	0.360
	Fe	0.321	−0.002	0.001	−1.506	0.005 *	0.000	0.000	−0.660
	Cr	0.321	−0.117	0.049	−2.732	0.032 *	−0.015	−0.001	−0.673
**BioT**	Mg	0.946	−0.004	0.005	0.180	0.002 *	−0.001	0.000	−0.546
	Mn	0.309	−5.291	12.942	1.164	0.001 *	0.673	2.064	0.902
	Zn	0.897	−0.066	0.060	−0.273	0.008 *	0.003	0.017	0.460
	Cu	0.257	−2.841	7.996	3.106	0.222	−0.196	0.812	0.365
	Fe	0.253	−0.056	0.020	−1.762	0.006 *	−0.008	−0.001	−0.647
	Cr	0.265	−2.767	1.006	−3.106	0.027 *	−0.341	−0.023	−0.696
**E2**	Mg	0.416	−0.088	0.173	1.749	0.133	−0.023	0.003	−0.325
	Mn	0.654	−312.939	220.115	−0.371	0.588	−22.973	39.880	0.225
	Zn	0.525	−2.312	1.385	−1.059	0.539	−0.230	0.431	0.128
	Cu	0.980	−156.928	159.906	0.047	0.607	−17.710	29.839	0.195
	Fe	0.963	−1.132	1.092	−0.050	0.916	−0.156	0.141	−0.044
	Cr	0.860	−58.897	51.417	−0.346	0.375	−10.772	4.171	−0.342

*p*—statistical significance; *—statistically significant parameter’ CI—confidence interval; BMI—body mass index; IIEF—International Index of Erectile Function; TT—total testosterone; FT—free testosterone; BioT—bioactive testosterone; E2—estradiol; Zn—zinc; Cu—copper; Fe—iron; Cr—chrome; Mg—magnesium; Mn—manganese.

## Data Availability

Data are available from the corresponding author.
